# More recent insights into the breast cancer burden across BRICS-Plus: Health consequences in key nations with emerging economies using the global burden of disease study 2019

**DOI:** 10.3389/fonc.2023.1100300

**Published:** 2023-01-24

**Authors:** Sumaira Mubarik, Lisha Luo, Mujahid Iqbal, Nawsherwan, Jianjun Bai, Chuanhua Yu

**Affiliations:** ^1^ Department of Epidemiology and Biostatistics, School of Public Health, Wuhan University, Wuhan, China; ^2^ Center for Evidence-Based and Translational Medicine, Zhongnan Hospital of Wuhan University, Wuhan, Hubei, China; ^3^ Department of Psychology, School of Philosophy, Wuhan University, Wuhan, China; ^4^ Xiamen Cardiovascular Hospital of Xiamen University, School of Medicine, Xiamen University, Xiamen, China

**Keywords:** breast cancer, mortality, case fatality, APC, SDI, BRICS-Plus

## Abstract

**Background:**

Brazil, Russia, India, China, South Africa, and 30 other Asian nations make up the BRICS-Plus, a group of developing countries that account for about half of the world’s population and contribute significantly to the global illness burden. This study aimed to analyzed the epidemiological burden of female breast cancer (BC) across the BRICS-Plus from 1990 to 2019 and studied the associations with age, period, birth cohort and countries’ sociodemographic index (SDI).

**Methods:**

The BC mortality and incidence estimates came from the 2019 Global Burden of Disease Study. We estimated cohort and period effects in BC outcomes between 1990 and 2019 using age-period-cohort (APC) modeling. The maximum likelihood (ML) of the APC-model Poisson with log (Y) based on the natural-spline function was used to estimate the rate ratio (RR). We used annualized rate of change (AROC) to quantify change over the previous 30 years in BC across BRICS-Plus and compare it to the global.

**Results:**

In 2019, there were about 1.98 million female BC cases (age-standardized rate of 45.86 [95% UI: 41.91, 49.76]) and 0.69 million deaths (age-standardized rate of 15.88 [95% UI: 14.66, 17.07]) around the globe. Among them, 45.4% of incident cases and 51.3% of deaths were attributed to the BRICS-Plus. China (41.1% cases and 26.5% deaths) and India (16.1% cases and 23.1% deaths) had the largest proportion of incident cases and deaths among the BRICS-Plus nations in 2019. Pakistan came in third with 5.6% cases and 8.8% deaths. Over the past three decades, from 1990 to 2019, the BRICS-Plus region’s greatest AROC was seen in Lesotho (2.61%; 95%UI: 1.99-2.99). The birth cohort impacts on BC vary significantly among the BRICS-Plus nations. Overall, the risk of case-fatality rate tended to decline in all BRICS-Plus nations, notably in South Asian Association for Regional Cooperation (SAARC) and China-ASEAN Free Trade Area (China-ASEAN FTA) countries, and the drop in risk in the most recent cohort was lowest in China and the Maldives. Additionally, there was a substantial negative link between SDI and case fatality rate (r_1990_= -0.91, *p*<0.001; r_2019_= -0.89, *p<*0.001) in the BRICS-Plus in both 1990 and 2019, with the Eurasian Economic Union (EEU) nations having the highest case fatality rate.

**Conclusions:**

The BC burden varies remarkably between different BRICS-Plus regions. Although the BRICS’ efforts to regulate BC succeeded, the overall improvements lagged behind those in high-income Asia-Pacific nations. Every BRICS-Plus country should strengthen specific public health approaches and policies directed at different priority groups, according to BRIC-Plus and other high-burden nations.

## Introduction

Breast cancer (BC) has now overtaken lung cancer as the most commonly diagnosed malignancy across the globe, with the majority of its health burden occurring in women (1). The GLOBOCAN 2020 estimates indicated that about 2.3 million new incidence cases and 685000 deaths were caused by breast cancer globally in 2020, accounting for 11.7% and 6.9% of total new cancer cases and deaths, respectively (2). The global burden of disease study 2019 (GBD 2019) estimated that the age-standardized incidence and mortality rates for females with breast cancer were 45.86 and 15.88 per 100,000 worldwide in 2019, respectively (3). Without new interventions, the BC burden will increase to more than 3 million new cases and more than 1 million deaths annually by 2040 (1).

Several internationally sustainable efforts have been made to improve the current situation of high burden of breast cancer, such as The Global Breast Cancer Initiative (GBCI), a strategic collaboration established by the World Health Organization in 2021, to control breast cancer burden by global advocacy (4). The GBCI aimed to the shared goal of reducing breast cancer by 2.5% per year, saving 2.5 million lives over 20 years by the three key strategies (health promotion; early detection and timely diagnosis; comprehensive breast cancer management) (4). Therefore, a better understanding of epidemiological characteristics of breast cancer burden across different regions/countries is of great value for providing a scientific foundation and experiences for policymakers to develop targeted strategies.

The previous studies have revealed that marked geographic discrepancies were observed in the BC burden, with the highest burden occurring in countries that underwent economic transition (1, 5). BRICS (Brazil, Russia, India, China, and South Africa) is an informal and financial group comprised of five countries with fast-economic transition, and BRICS-Plus is a new economic group to bring together the regional integration blocks where BRICS economies play a leading role, which was made up by 35 developing countries “in transition” (6). Therefore, it is urgent to investigate the patterns of breast cancer burden in BRICS Plus countries to explore the underlying causes of these disparities using advanced statistical models, thereby helping policymakers tackle this public health challenge and better promote health and economic integration. Additionally, the Socio-Demographic Index (SDI) has been considered an effective measure for predicting and comparing regional and national burdens of disease, evaluating the effectiveness of health policies in countries with socioeconomic transition (7). A previously published review showed there were 33.6% of global new cases and 36.9% of global deaths of breast cancer occurred in BRICS countries, and a marked rising trend in incidence was also observed in these countries, but the corresponding epidemiological variations of its burden and its correlation to age group, birth cohort, and socioeconomic status are not well studied (8).

The current study aimed to compare the long-term trends in incidence, mortality and case fatality rates of breast cancer in BRICS -Plus countries and at the global level, investigating the independent effects of age, period and cohort using data from the Global Burden of Disease Study 2019 (GBD 2019) within the age-period-cohort (APC) framework, and exploring the potential causes of the associations between SDI and these health outcomes. The findings of this study give a combined comparison of breast cancer burden across BRICS Plus countries, which may provide references for the rational allocation of healthcare resources and help policymakers to specify prevention and control strategies according to local conditions.

## Material and methods

All anonymized data are accessible online at http://ghdx.healthdata.org/gbd-results-tool, the Institute for Health Metrics and Evaluation. The deidentified, compiled data was used in the GBD investigation. As a result, the University of Washington Institutional Review Board examined and approved a waiver of informed consent.

### Data source, estimation of study variables, and study population

The most comprehensive and systematic epidemiological study to date is called the Global Burden of Diseases (GBD). It offers a comprehensive analysis of 359 illnesses and injuries, 84 risk factors, and 282 recorded causes of mortality across 195 nations, 21 regions, and seven super-regions. The GBD uses novel estimation techniques and supporting sources specified in earlier research to give the 2019 estimates and revisions for 1990-2019 data (9–12). The Global Health Data Exchange (GHDx) online data collecting tool (http://ghdx.healthdata.org/gbd-results-tool) has been used to gather data on breast cancer (BC). The “Availability of data and materials” section at the article’s end defines all the data sources’ specifics. From 1990 to 2019, gathered information on BC from 35 BRICS -Plus nations’ yearly death and incidence rates and age-standardized rates (ASRs) per 100k person-years. The sociodemographic index (SDI), which is based on national per capita income, the average number of years of education for those over 15 years old, and the overall fertility rate, was developed to divide countries into five quintiles (high SDI, high middle SDI, middle SDI, low middle SDI, low SDI). From 0 to 1, or from least to most developed, is the range of this index.

The GBD study states that estimates of BC incidence are based on integrated cancer registry databases or individual cancer registries. The percentage of data gathered from the systematic literature study was subjected to four separate DisMod-MR2.1 inputs (13). As the primary method for estimating the prevalence and incidence of 354 injuries and diseases in 195 nations and territories, DisMod-MR2.1 is based on the Bayesian meta-regression tool.

The BRICS-Plus countries were divided into five regions, including Mercosur (core members as well as acceding members), South African Customs Union (SACU), SAARC (SAFTA members), China-ASEAN FTA and Eurasian Economic Union (EEU), and the specific subdivision list of 35 countries was as follows: Mercosur (Brazil, Argentina, Paraguay, Uruguay, Bolivia, Venezuela); SACU (Botswana, Lesotho, Namibia, South Africa, Swaziland); SAARC (Afghanistan, Bangladesh, Bhutan, India, the Maldives, Nepal, Pakistan, Sri Lanka); China-ASEAN FTA (China, Indonesia, Malaysia, Philippines, Singapore, Thailand, Brunei, Vietnam, Laos, Myanmar, Cambodia); EEU (Russia, Kazakhstan, Belarus, Armenia, Kyrgyzstan).

This study’s female population was diagnosed with BC using the ICD-10 code C50 for the disease (14). The population estimates from 1990–2019 from the online Global Health Data Exchange (GHDx) were used to calculate the matching population estimates for the BRICS-Plus Countries by age group (20-24 to 80-84) and year (1990 to 2019) (15). For instance, the original data for Brazil came primarily from the Ministry of Health’s Mortality Information System (16); New Economic School’s Russian Centre for Demographic Research provides mortality data by age, sex, location, and cause of death (17); The mortality database for India is managed by the Registrar General of India and the Indian Sample Registration System (18); The Chinese Center for Disease Control and Prevention, Disease Surveillance Points, Maternal and Child Surveillance System, and Cause of Death Reporting System are in charge of maintaining the health statistics database for the Chinese population (19). The Department of Home Affairs South Africa Vital Registration system is responsible for keeping track of the country’s overall mortality statistics by age, sex and region and cause of death. Other BRICS-Plus countries used their current health database registration systems to estimate the BC figures. Due to the lack of information on various diseases, injuries, and risk factors from various countries, estimates from GBD rely on complex statistical modeling to handle sparse and usually inconsistent data (20). CODEm (Death Ensemble Model) was run using mortality projections and vital registration system data for BC (21). Based on information and factors, such as education, SDI, lagging distribution income, smoking habit and alcohol consumption, the CODEm calculates mortality rates. The single-cause estimates have been modified to suit all-cause mortality separately using the CodCorrect technique (22, 23). The series of publications written by GBD include specific computation methodologies. In the collection of articles released by GBD, specific calculating techniques are mentioned (11, 24–26).

The GBD approach has the advantage of using consistent methods to critically evaluate the currently available data on each condition, making this data comparable and systematic; estimating results from countries with incomplete data; and reporting on the burden of disease with standardized metrics.

### Statistical analysis

We investigated the geographic assessment of BC changes by adopting a graphical display and stratifying by time and age group. We presented annualized rates of change (AROC) for changes over time as the proportional difference between the rate’s natural logarithms in 1990 and 2019 divided by 29 (i.e., 100*[ln(2019 Rate/1990 Rate)/(29)]). AROC (%) is a crude trend measure over a 29-year period. An increasing trend/slope or acceleration of mortality/incidence over 29 years in BC is indicated by a positive AROC. In contrast, a decreasing trend/slope or deceleration is shown by a negative AROC. Additionally, based on one thousand bootstrap draws from the posterior distribution, uncertainty intervals (UIs) were used to quantify uncertainty for each outcome (21, 27). We calculated Point estimates from the mean, and UIs were calculated using the 25th and 975th ordered values of the posterior distribution of the 1000 drawings. When the 95% UI of the percentage change did not contain zero, changes over time were deemed statistically significant. For 35 BRICS-Plus nations and globally, we estimated the percent change in age-standardized BC mortality and incidence rates between 1990 and 2019. We gave the 2019 estimates with 95% uncertainty intervals (UI) for those 35 countries. By dividing the age-standardized death rate (ASDR) by the age-standardized incidence rate (ASIR), then multiplying the result by 100, the case-fatality percent (CFP) was determined (28). Spearman correlation coefficient(r) was used to examine the relationship between SDI and BC outcomes.

The relationship between several BC-related indicators, including incidence, mortality, and case fatality rate, and each of the three primary sources of spatial and temporal variability—age, period (year), and cohort (year of birth)—was examined for each BRICS-Plus region/country as well as globally within the framework of the age-period-cohort model. Period effects show population-wide exposure at a specific moment, and the period was defined as the survey year. The term “cohort effects” refers to variations in hazards among birth cohorts. Age-specific rates of BC from 20 to 84 years with subsequent 5-year age intervals, calendar time including a consecutive period from 1990 to 2019, and subsequent cohort (period-age) from 1905 to 1999 were taken into consideration for statistical analysis in the age-period-cohort study. Using the R package (Epi, version 2.44) created by Carstensen et al.,2021, we carried out the APC analysis (29). Maximum Likelihood (ML) of the APC-model Poisson with log (Y) based on the natural-spline function was used to estimate the rate ratio. The reference cohort and reference period were respectively chosen based on the median date of birth and the median date of diagnosis among cases. The estimated models’ deviation table was used to assess the goodness of fit. To assess the study hypothesis, two-sided statistical tests were taken into consideration. The threshold for statistical significance of the findings was set at 1% (level of significance).

## Results

### Burden and trends in female BC by BRICS-Plus regions and globally

**Figure 1:**
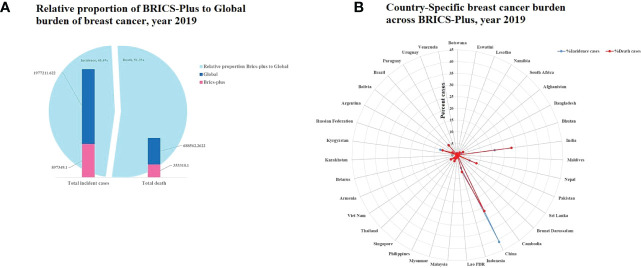
**(A)** Relative proportion of BRICS-Plus to Global burden of breast cancer incidence and death in year 2019, **(B)** Country specific breast cancer burden within BRICS-Plus; percent cases (incidence and death) indicate proportion of country specific cases to total cases in BRICS-Plus.

**Figure 1** and **Supplementary Figures S1, S2** show the burden and trends in female breast cancer (BC) mortality and incidence across the BRICS-Plus countries and globally from 1990 to 2019.

In 2019, there were about 1.98 million female BC cases (age-standardized rate of 45.86 [95% UI: 41.91, 49.76]) and 0.69 million deaths (age-standardized rate of 15.88 [95% UI: 14.66, 17.07]) around the globe. Among these, 45.4% of incident cases and 51.3% of deaths contributed to overall BRICS -Plus. Across the BRICS -Plus countries, there were the highest proportion of incident cases and deaths from China (41.1%cases and 26.5%deaths) and India (16.1%cases and 23.1%deaths), followed by Pakistan (5.6% cases and 8.8%deaths) in 2019. Overall the trends in case fatality percent (CFP) declined across the BRICS -Plus from 1990 to 2019, but these trends were higher than global CFP. The highest CFP was observed in Lesotho during the entire duration from 1990 to 2019 than in other BRICS-Plus countries (**Figures 1**, **S1, S2**).

### The annualized rate of change in BC over 30 years across BRICS-Plus and globally

**Figure 2:**
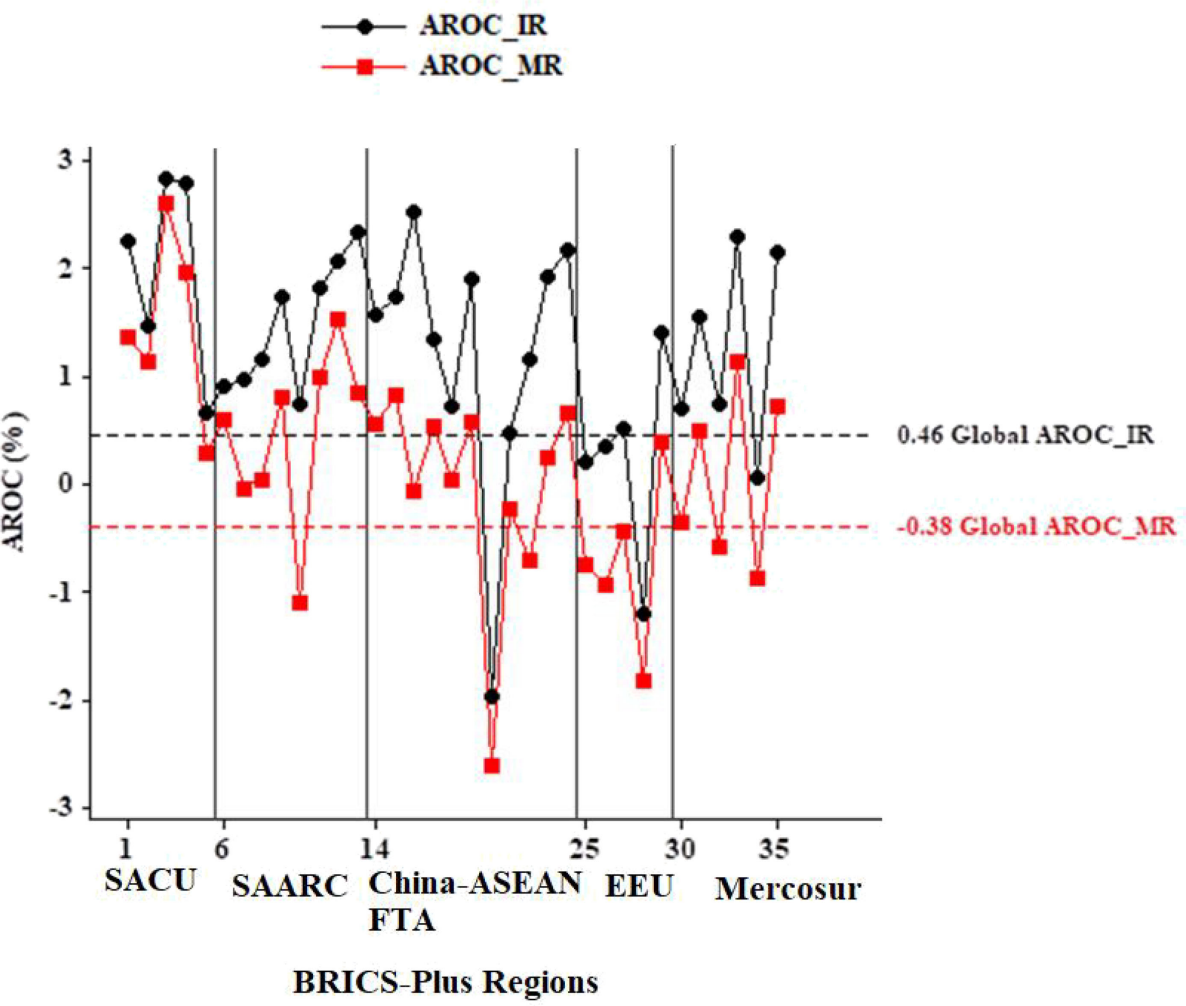
Comparison of annualized rate of change (AROC) BRICS-Plus to Global burden of breast cancer. IR, Incidence rate; MR, Mortality rate.

Compared to the other BRICS-Plus regions, the SACU’s BC age-standardized mortality rate (ASMR) grew by 7.38% (%AROC Range: 0.29-2.61) from 1990 to 2019. Lesotho had the highest AROC (%) among the BRICS-Plus countries from 1990 to 2019 (2.61% (95%UI: 1.99,2.99). Thirteen of the 35 BRICS-Plus nations, divided into the SACU, SAARC, China-ASEAN FTA, EEU, and Mercosur regions, had a decline in ASMR over the same period. Only 4 of these nations’ ASMR changes were less than the worldwide AROC. In addition, all of the BRICS-Plus countries saw a rise in the AROC for age-standardized incidence rate (ASIR), except for Myanmar and Kyrgyzstan, where ASIR decreased by 1.98% (95%UI: -1.53, -2.29) and 1.19% (95%UI: -1.48, -0.94) over the past three decades from 1990 to 2019. From 1990 to 2019, all of the BRICS-Plus countries had great changes relative to the global AROC in ASIR (**Table 1** and **Figure 2**).

**Table 1:** Age-standardised breast cancer mortality rate and incidence rate (per 100 000 person-years) and Annualized rate of change (AROC%), 1990 to 2019 across BRICS-Plus regions.

	Age-standardised mortality rate (per 100,000 person-years)			Age-standardised incidence rate (per 100,000 person-years)		
	1990_MR (95%UI)	2019_MR (95%UI)	%AROC (95%UI)	1990_IR (95%UI)	2019_IR (95%UI)	%AROC (95%UI)
Global	17.76 (16.93,18.51)	15.88 (14.66,17.07)	-0.38 (-0.28, 0.5)	40.12 (38.78,41.33)	45.86 (41.91,49.76)	0.46 (0.27,0.64)
SACU						
Botswana	19.28 (14.11,26.16)	28.63 (19.05,41.04)	1.36 (1.03,1.55)	25.43 (18.3,35.04)	49.02 (31.66,72.18)	2.26 (1.89,2.49)
Eswatini	16.55 (11.84,21.09)	23.03 (14.59,33.51)	1.14 (0.72,1.6)	20.67 (14.71,26.35)	31.61 (19.22,47.07)	1.46 (0.92,2.0)
Lesotho	13.29 (10.17,17.55)	28.37 (18.11,41.78)	2.61 (1.99,2.99)	16.21 (12.19,21.64)	37 (23.32,55.81)	2.85 (2.24,3.27)
Namibia	16.73 (12.73,20.9)	29.68 (20.88,41.74)	1.98 (1.71,2.39)	21.34 (16.03,26.84)	48.17 (33.07,70.08)	2.81 (2.5,3.31)
South Africa	18.83 (16.34,22.12)	20.48 (18.08,23.22)	0.29 (0.17,0.35)	26.48 (23.4,30.4)	32.08 (27.82,36.95)	0.66 (0.60,0.67)
SAARC						
Afghanistan	13.86 (10.99,17.5)	16.47 (12.51,21.29)	0.59 (0.45,0.68)	17.05 (13.36,21.73)	22.28 (16.83,29.07)	0.92 (0.8,10)
Bangladesh	14.74 (11.1,18.71)	14.54 (11.51,18.15)	-0.05 (-0.11,0.12)	18.89 (14.26,24.15)	25.03 (19.56,31.81)	0.97 (0.95,1.09)
Bhutan	11.84 (7.83,16.69)	12.02 (8.53,16.35)	0.05 (0.07,0.29)	14.91 (9.77,21.21)	20.87 (14.61,28.78)	1.16 (1.05,1.39)
India	10.81 (8.77,12.75)	13.67 (10.57,17.35)	0.81 (0.64,1.06)	13.93 (11.3,16.5)	23.04 (17.79,28.97)	1.74 (1.57,1.94)
Maldives	18.74 (11.95,26.73)	13.65 (11.14,16.51)	-1.09 (-1.66,-0.24)	27.23 (16.59,39.96)	33.75 (26.91,40.94)	0.74 (0.08,1.67)
Nepal	13.55 (8.75,18.84)	18.08 (13.41,23.83)	0.99 (0.81,1.47)	16.94 (11.13,23.71)	28.81 (21.2,38.35)	1.83 (1.66,2.22)
Pakistan	33.18 (23.25,46.34)	51.94 (39.03,69.76)	1.54 (1.41,1.79)	41.83 (29.52,58.19)	76.49 (56.07,102.5)	2.08 (1.95,2.21)
Sri Lanka	9.48 (8.21,10.79)	12.14 (8.99,16.07)	0.85 (0.31,1.38)	15.07 (13.08,17.2)	29.78 (21.86,40.03)	2.35 (1.77,2.91)
China-ASEAN FTA						
Brunei Darussalam	19.5 (16.15,23.48)	22.9 (19.26,27.04)	0.55 (0.49,0.61)	42.86 (34.73,52.41)	67.73 (55.46,82.13)	1.58 (1.55,1.61)
Cambodia	10.87 (8.12,14.07)	13.86 (10.73,17.11)	0.84 (0.67, 0.96)	14.15 (10.66,18.54)	23.52 (17.91,29.43)	1.75 (1.59,1.79)
China	9.16 (7.61,10.82)	9.02 (7.19,11.1)	-0.06 (-0.2,0.09)	17.07 (14.02,20.3)	35.61 (28.07,44.81)	2.54 (2.39,2.73)
Indonesia	17.48 (14.98,20.57)	20.47 (15.89,25.94)	0.55 (0.2,0.8)	25.38 (21.76,29.96)	37.42 (28.96,48.59)	1.34 (0.99,1.67)
Lao People’s Democratic Republic (Lao PDR)	20.55 (14.05,30.05)	20.79 (15.1,29.08)	0.04 (-0.11,0.25)	25.32 (16.91,37.58)	31.24 (22.4,44.28)	0.73 (0.57,0.97)
Malaysia	21.87 (19.35,25.06)	25.81 (19.83,32.54)	0.57 (0.08,0.9)	34.14 (30.25,38.88)	59.48 (45.19,75.15)	1.91 (1.38,2.27)
Myanmar	35.73 (26.13,48.22)	16.78 (13.87,20.84)	-2.61 (,-2.89,-2.18)	47.27 (33.3,65.1)	26.64 (21.39,33.54)	-1.98 (-2.29,-1.53)
Philippines	24.48 (21.37,27.41)	22.85 (17.39,29.48)	-0.24 (-0.71,0.25)	35.45 (30.84,40.23)	40.63 (30.52,52.96)	0.47 (-0.04,0.95)
Singapore	15.51 (14.64,16.37)	12.66 (11.44,13.77)	-0.7 (-0.6,-0.85)	44.68 (41.25,48.67)	62.72 (49.6,78.06)	1.17 (0.64,1.63)
Thailand	11.54 (10.04,13.27)	12.41 (9.08,16.35)	0.25 (-0.35,0.72)	19 (16.37,22.01)	33.31 (23.94,44.6)	1.94 (1.31,2.44)
Viet Nam	17.74 (14.09,21.85)	21.54 (16.39,27.78)	0.67 (0.52,0.83)	25.84 (20.1,32.26)	48.65 (36.57,63.02)	2.18 (2.06,2.31)
EEU						
Armenia	29.64 (28,31.28)	23.95 (19.86,28.52)	-0.74 (-1.18,-0.32)	57.5 (53.82,61.4)	61.17 (49.78,73.72)	0.21 (-0.27,0.63)
Belarus	18.35 (17.53,19.25)	14.01 (10.77,18.08)	-0.93 (-1.68,-0.22)	44.19 (41.25,47.03)	48.88 (36.92,64.83)	0.35 (-0.38,1.11)
Kazakhstan	18.45 (17.25,19.67)	16.3 (13.96,18.85)	-0.43 (-0.73,-0.15)	34.42 (31.86,36.99)	40.07 (33.62,47.05)	0.52 (0.19,0.83)
Kyrgyzstan	18.33 (17.13,19.82)	10.8 (9.36,12.47)	-1.82 (-2.08,-1.6)	31.71 (29.34,34.43)	22.43 (19.09,26.21)	-1.19 (-1.48,-0.94)
Russian Federation	15.14 (14.71,15.73)	17.02 (13.91,20.46)	0.4 (-0.19,0.91)	36.08 (35.09,37.72)	54.39 (44.01,66.87)	1.42 (0.78,1.97)
Mercosur						
Argentina	30.92 (29.62,32.19)	27.92 (25.85,30.04)	-0.35 (-0.47,-0.24)	51.04 (48.7,53.51)	62.72 (48.36,80.34)	0.71 (-0.02,1.4)
Bolivia	16.45 (13,21.08)	18.98 (14.75,24.35)	0.49 (0.44,0.50)	21.98 (17.08,28.22)	34.52 (26.16,45.81)	1.56 (1.47,1.67)
Brazil	17.87 (17.07,18.53)	15.13 (14.1,16.08)	-0.57 (-0.66,-0.49)	31.95 (30.6,33.06)	39.64 (37.1,42.28)	0.74 (0.66,0.85)
Paraguay	12.94 (11.06,14.92)	18.04 (13.71,23.01)	1.15 (0.74,1.49)	22.77 (19.44,26.53)	44.33 (32.75,57.04)	2.3 (1.8,2.64)
Uruguay	38.52 (36.58,40.12)	29.97 (27.54,32.27)	-0.87 (-0.98, -0.75)	71.14 (66.32,75.9)	72.65 (55.79,92.57)	0.07 (-0.60,0.68)
Venezuela	14.29 (13.46,15.02)	17.65 (13.43,23.06)	0.73 (-0.01,1.48)	28.44 (26.72,30.37)	53 (39.43,70.51)	2.15 (1.34,2.9)

### BC age-specific mortality rates across BRICS-Plus and globally

**Figure 3:**
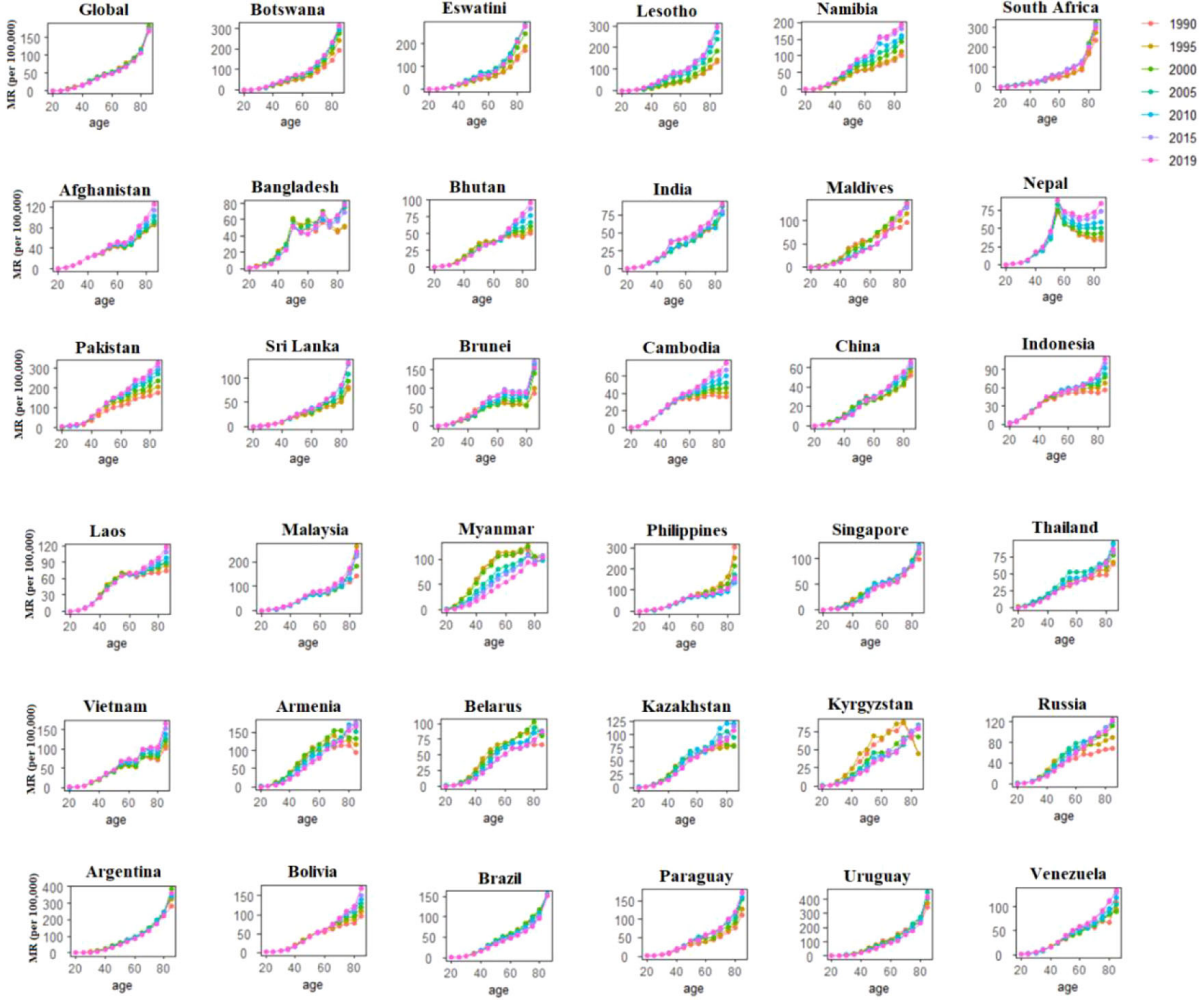
Age-specific mortality rates of breast cancer by period across 35 BRICS-Plus countries and Globally between 1990 to 2019.

We organized the population and BC (mortality and incidence) data into 19 consecutive cohorts, including those born from 1905 to 1909 (median, 1907) through 1995 to 1999 (median, 1997) and consecutive 5-year periods from 1990 to 1994 (median, 1992) to 2015 to 2019 (median, 2017). **Figure 3** displays the BC mortality rate trends by age group over time in the BRICS-Plus and globally. The findings show that the BC mortality rate has risen over time in most of the BRICS-Plus regions, with age group. At the same time, recent years saw the most significant reductions in BC mortality in Myanmar, the Philippines, Singapore, Thailand, Armenia, Belarus, Kazakhstan, and Kyrgyzstan (*P*<0.01 for all) (**Figure 3**).

**Figure 4:**
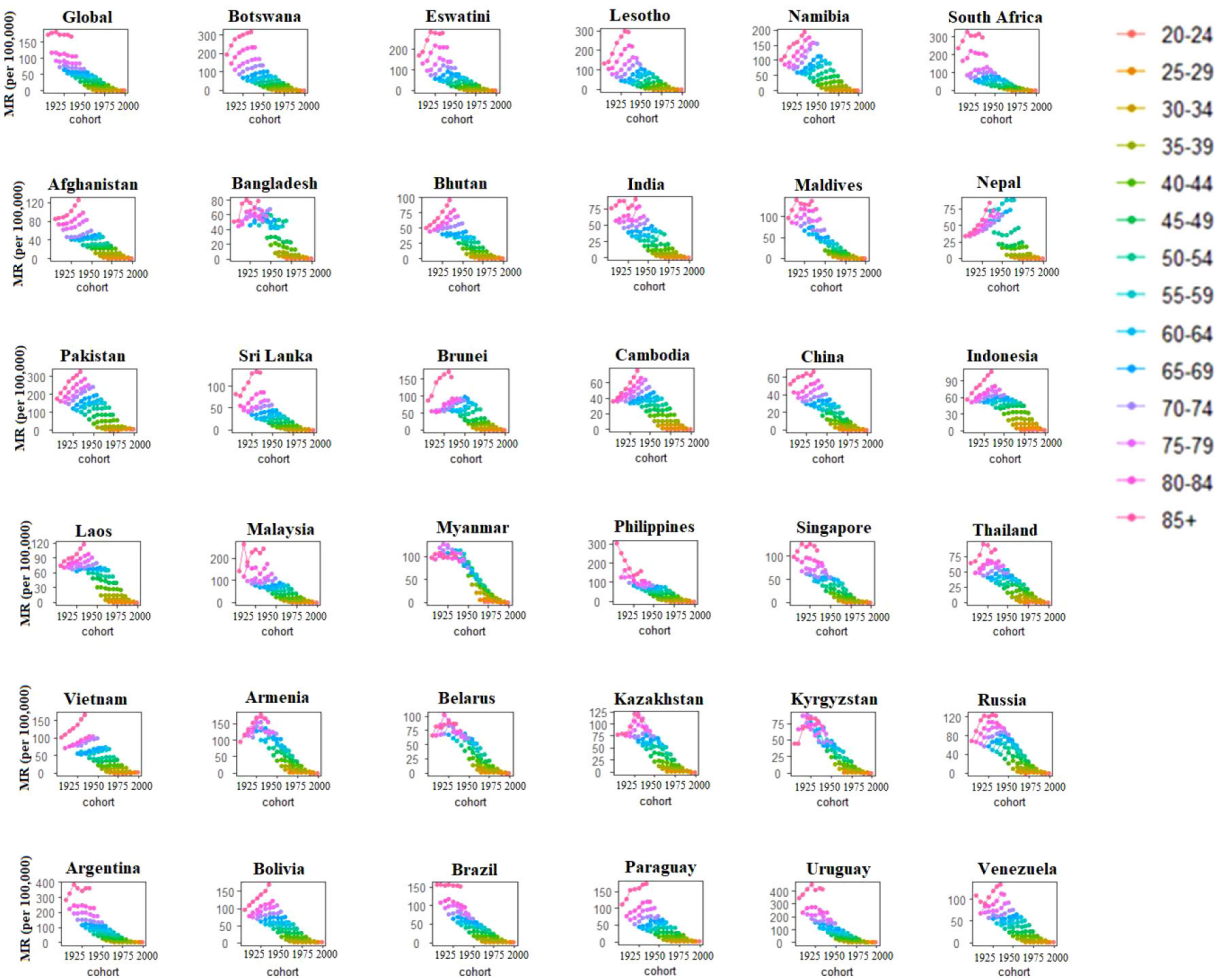
Cohort-specific mortality rates of breast cancer by age group across 35 BRICS-Plus countries and Globally between 1990 to 2019.

From **Figure 4**, we can observe that globally there was a decreasing trend in BC mortality across the birth cohorts. In contrast, most of the BRICS-Plus countries showed throughout increasing trend of BC mortality rate across birth cohorts. Eswatini, South Africa, Bangladesh, India, Maldives, Sri Lanka, Malaysia, Philippines, Singapore, Thailand, Armenia, Belarus, Kazakhstan, Kyrgyzstan, Russia, Argentina, and Uruguay all displayed an increase in BC mortality rates at first, followed by a decline across all age groups (*p<*0.001 for all). For instance, those under 50 had consistently lower BC mortality over subsequent birth cohorts than those over 50 (**Figure 4**).

### Age period cohort effects on BC across BRICS-Plus and globally

**Figure 5:**
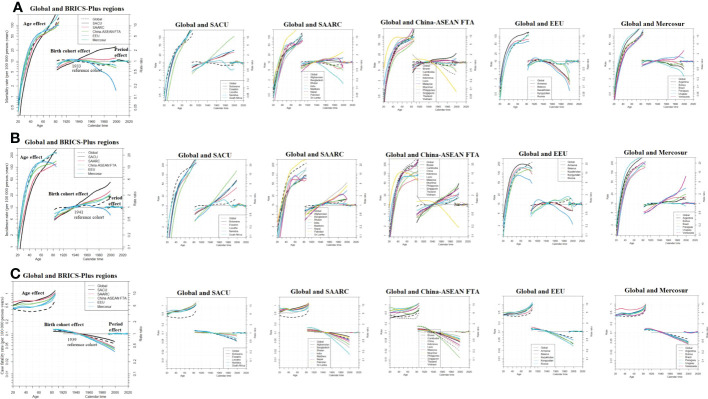
Age period cohort related female breast cancer trends in **(A)** death rate **(B)** incidence rate and **(C)** case fatality rate (CFR) from 1990-2019 with ages 20 to 84. Rate ratio was estimated using ML of APC-model Poisson with log**(Y)** based on natural-spline function, for each BRICS-Plus region separately. ML, maximum likelihood; APC, age-period-cohort; Reference cohort for age-effects was chosen as the median date of birth among cases; and Median date of diagnosis among cases was selected as reference period; SACU, South African Customs Union; SAARC, South Asian Association for Regional Cooperation; China-ASEAN FTA, China-ASEAN Free Trade Area; EEU, Eurasian Economic Union; Mercosur, core members as well as acceding members.

To account for the age, period, and cohort influences of BC outcome in each BRICS-Plus region and country, we computed the APC trends in BC mortality, incidence, and case fatality rates. **Figure 5** illustrates these effects while keeping all other variables fixed at their sample mean values.

For each BRICS-Plus region, age-related effects can be seen to have a significant impact on BC mortality. The SACU, SAARC, and Mercosur regions show the most striking increases in BC mortality risk, with Botswana, Eswatini, South Africa, Pakistan, Uruguay, and Argentina having the highest age-related risk countries.

We found birth cohort impacts vary significantly among the BRICS-Plus nations. In the majority of BRICS-Plus countries, the study revealed an increase in mortality risk trend when birth cohorts increased from the cases’ median birthdate. Except for the EEU and a few other countries from other regions, the risk of death was rising in all BRICS-Plus regions. Lesotho, Pakistan, Burnie, and Venezuela stood out as high cohort-risk nations (**Figure 5A**). The risk of BC mortality rate tends to rise with age and cohort, and the risk value was more significant than the global effect.

The age period cohort effects on the incidence rate of BC are shown in **Figure 5B**. Except for the EEU, where a downward trend was seen after the birth cohort of 1945, all BRICS-Plus regions saw an increase in overall age and cohort-associated incidence risks. Most of the nations in the SACU, SAARC, China-ASEAN FTA, and Mercosur regions had higher risk values than the globe. In particular, Lesotho, Sri Lanka, Vietnam, and Venezuela exhibited high incidence cohort-related risks. For cohorts older than 50 and those born between 1940 and after, the effect of separation across different BRICS-Plus regions was more pronounced. In SAARC and Mercosur, the period impact persisted over the entire period. In contrast, SACU, China-ASEAN FTA, and EEU showed moderate changes during the whole study period from 1990 to 2019 (**Figure 5**).

To fully understand the BC burden, we also did an APC analysis on case-fatality rates (CFR) by BRICS-Plus regions and nations. The Maldives and China experienced the smallest decrease in the risk of case-fatality rate in the most recent cohort, as illustrated in **Figure 5C**. In the BRICS-Plus region as a whole, case fatality rates tend to drop, particularly in the SAARC and China-ASEAN FTA countries. Between different BRICS-Plus regions, the effect of separation was more evident for cohorts beginning in 1939 and later for those who were greater than 40 years older. In the majority of BRICS-Plus nations and throughout the world, the period impact persisted for the whole time (**Figure 5C**).

### Relationship between sociodemographic index and BC incidence, mortality and case-fatality across BRICS-Plus

**Figure 6:**
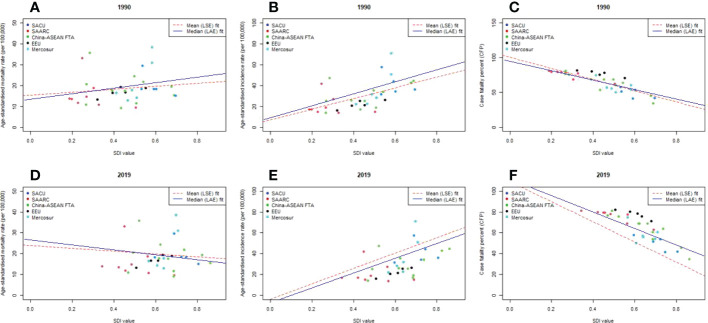
Country wise correlation between age-standardised BC rate and country’s sociodemographic index (SDI) across BRICS-Plus; **(A)** mortality rate in 1990 **(B)** incidence rate in 1990 **(C)** Case fatality percent (CFP) in 1990; **(D)** mortality rate in 2019 **(E)** incidence rate in 2019 **(F)** Case fatality percent (CFP) in 2019; Case-fatality percent was calculated by dividing age-standardised death rate by age-standardised incidence rate and multiplied by 100; LSE, Least Square Error fit; LAE, Least Absolute Error fit; SDI ranges from 0 (less developed) to 1 (most developed).

**Figure 6** illustrates the bivariate relationship between country-wise age-standardized mortality rates, incidence and case fatality percent (CFP) and countries’ SDI for the years 1990 and 2019. We can see that while ASMR and SDI showed mixed patterns throughout BRICS-Plus, for example, the nations that improved their SDI in 2019 also decreased ASMR, ASIR established a positive and statistically significant link with SDI in both 1990 and 2019. Additionally, there was a substantial negative link between SDI and CFP (r_1990_= -0.91, *P*<0.001; r_2019_= -0.89, *P*<0.001) in the BRICS-Plus region in both 1990 and 2019, with the EEU nations having the highest case fatality rates.

Additionally, different patterns of epidemiological variations were seen in **Figure S3** when age-standardized mortality rate (ASMR), age-standardized incidence rate (ASIR), and case fatality percent (CFP) in each BRICS-Plus country from 1990 to 2019 were plotted against an index of that country’s sociodemographic status in the same year. Along with SACU, SAARC, China-ASEAN FTA, and Mercosur, the ASIR of BC increased with rising SDI in different years, but the ASMR has shown notable variations. As SDI rises, ASIR gradually grows, with more rapid growth for the nations with the greatest levels of SDI. Most of the BRICS-Plus countries showed a downward trend in ASMR, especially those with high SDIs, which had a progressive decline in ASMR as SDI rose. Lesotho, Pakistan, and Bolivia, in contrast to certain BRICS-Plus nations with poor SDI, have had strong or consistent trends in ASMR over time. Additionally, from 1990 to 2019, across the entire BRICS-Plus region, a significant negative association between case fatality percentage and SDI was seen (**Figure S4**).

## Discussion

Breast carcinogenesis has been widely considered a significant global public health concern that continues to increase due to genetic, environmental, lifestyle and socioeconomic risk factors (30). The APC framework allowed estimations of when and how three time-dependent parameters would affect the variations of disease burden, and its result was helpful for theorizing the etiology of the observed trends (31). The current study has examined the age, period and cohort effects of breast cancer incidence, mortality and case fatality rates among females and evaluated the association of these health outcomes with sociodemographic transition across 35 BRICS-Plus countries based on GBD 2019 data from 1990 to 2019. The results revealed that nearly half of global incidence and deaths of breast cancer contributed from BRICS-Plus countries, and its case fatality percent was also higher than the worldwide level, which was consistent with previous studies indicating a shift in the BC burden from high-income to middle- and low-income countries (30, 32, 33). The high proportion suggested that the breast cancer burden was still a serious public health concern in BRICS-Plus countries, so more attention should be paid to exploring the underlying causes of this situation.

Within the BRICS-Plus, striking disparities in breast cancer burden were also observed among these 35 developing countries, with a higher proportion of incidence and deaths in China, India and Pakistan, mainly due to the large population. China, India and Pakistan are among the top five countries in the world with the largest population, with China home to about 1.45 billion, India to 1.40 billion and Pakistan to 0.23 billion in 2022 (34). The total population of China and India account for 36% of the world population, and Pakistan has the highest population growth between 2000 and 2022 (34). Population awareness, healthcare conditions, lifestyles, and socioeconomic factors also played an essential role in the higher cases in these three countries (5, 31). The case fatality percent (CFP) of breast cancer showed decreasing trends across the BRICS-Plus from 1990 to 2019, which indicated the improvement in the quality of healthcare, better prognosis (earlier detection and advanced therapy) and increasing survival rates of cancer cases in these countries (35). Across the BRICS-Plus countries, the highest CFP of breast cancer occurred in Lesotho, a small landlocked country in Southern Africa. It was highly associated with its backward healthcare systems, shortage of health resources and unequal service delivery (36). As we know, Lesotho is one of the most underdeveloped countries declared by the United Nations, which suffers from higher poverty rates, with 72% of the population living in rural areas far away from healthcare services and having experienced an increasing double disease burden from communicable diseases as well as non-communicable diseases.

Additionally, Lesotho was one of 28 African countries that didn’t provide radiotherapy, so many patients cannot be diagnosed and treated early. Although some policies have been made for its health systems challenges in Lesotho, such as the “5-year National Strategic Development Plan (NSDP)”, more effective and targeted measures are also urgent to curb the severe situations in this country (36). Specifically, the most urgent need for Lesotho is adopting radiotherapy for cancer treatment and providing mammographic screening for females above the age of 50 to improve the early diagnosis and treatment of breast cancer and effectively reduce the high case fatality of breast cancer in this country.

The significantly increasing trends in incidence and mortality of breast cancer were observed in most of the 35 BRICS-Plus countries over 30 years, which indicated the increasing burden of breast cancer from developed countries to developing countries confirmed in the prior studies (1, 30). From our results, the SACU region had the largest increase in breast cancer mortality and incidence over the last three decades, which were also consistent with other studies and mainly attributable to the late-stage prognosis and inadequate access to high-quality healthcare resources (1, 37). Additionally, genetic factors also played a key role in the growing incidence of breast cancer in the SACU region, and it’s confirmed the variants in the two major genes, BRCA1 and BRCA2, and in five other genes in the patients in South Africa (38). The incidence increase in most of the BRICS-Plus countries was also associated with mammographic screening, diet changes, improved health awareness, and reduced physical activity (30). The stage at diagnosis was a key prognostic factor for mortality of breast cancer, and a previous meta-analysis had revealed that about 77% of all cases were stage III and IV at diagnosis across 17 sub-Saharan African countries, while only 15% of cases were diagnosed at stages III and IV in some high-income countries (1, 39). Even though the incidence and mortality showed decreasing in a few countries, the AROC in most BRICS-Plus countries were still higher than the global level, so the overall condition of breast cancer in BRICS-Plus still lagged behind those in developed Asia Pacific countries, especially in SACU region. Recognizing this, the BRICS-Plus countries, especially high-risk regions, should learn and translate the experiences from developed countries into strategies suited to their characteristics, including early screening and treatment, improvement of population awareness, and healthier lifestyle, to strengthen the prevention and intervention of breast cancer in high-risk regions, and reduce the public health burden caused by breast cancer.

Age has been considered one of the most important and independent risk factors for developing breast cancer (40), and its incidence and mortality varied across the different age groups in BRICS-Plus countries. After adjusting the influence of period and cohort effect using the APC analysis, the BC mortality and incidence risk showed increasing trends with advancing age, and the older women suffered higher chances, which was consistent with the age trends of breast cancer in the previous studies (31, 41, 42). DNA methylation might be one of the important reasons behind the increased burden with age; additionally, biological factors and hormone levels were also well-proven factors for breast cancer (42, 43). The cohort effects revealed various exposures to behavioral, environmental and socioeconomic factors among different birth cohorts, and our results indicated that the incidence and mortality risk increased with the birth cohorts in most of the BRICS-Plus countries, which indicated that the later birth cohorts had higher risks of breast cancer than earlier cohorts in these countries. On the contrary, the risk of case-fatality rate showed decreasing trends revealing earlier birth cohorts suffered from a higher risk of the case-fatality rate. The previous study also revealed that the risk for breast cancer increased slightly in low-middle and low-socioeconomic regions. At the same time, decreasing trends were observed in the high-socioeconomic areas, which was consistent with our results (30). The possible reasons behind the increasing cohort effects in most countries were westernized lifestyles, inequality of diagnosis, prognosis and treatment of breast cancer across BRICS-Plus countries, and it also highlighted that the improvement of diagnostic tools and easy access to treatment helped decrease breast cancer burden (30, 44). The period effects remained consistent for BC case-fatality rates across most of the BRICS-Plus countries during the entire duration. In contrast, incidence and mortality remained constant in SAARC and Mercosur regions. In contrast, they showed moderate fluctuations in SACU, China-ASEAN FTA and EEU regions, which reflected the immediate effects of the risk factors on breast cancer burden adjusted for age and cohort effects.

In our study, an increase of SDI tended to be positively associated with an increase of breast cancer incidence, which was also confirmed in the previous studies (30, 45). The positive association between socioeconomic status and breast cancer incidence might be caused by easier access to early detection and screening measures in countries with higher socioeconomic status; besides, drastic changes in lifestyle, environmental factors and the use of hormone replacement therapy also played a crucial role (45). The lower incidence of breast cancer in countries with lower socioeconomic status might be underestimated because of unreliable disease surveillance and reporting systems and lower screening rates (45). Therefore, less developed regions should strengthen screening efforts and improve disease surveillance systems to reflect local disease burden levels more accurately. The declining trends of the relationship between case fatality burden and SDI in 1990 and 2019 across the BRICS-Plus were also consistent with the results conducted in 21 world regions, which indicated better treatment facilities in high-income regions and limited treatment resources in less developed regions (30). The mixed patterns of the relationship between mortality and SDI revealed the shift in the death burden of breast cancer from developed to less developed countries (30).

The study also had several limitations. Firstly, all data about breast cancer in our study were obtained from GBD 2019. Some data were not directly measured when the reliably original data was lacking or reporting lag. Hence, it didn’t remain very objective or ambiguities (46). Secondly, interpreting results from the age-period-cohort model at the population level doesn’t necessarily hold for individuals, so there might be an ecological fallacy. Therefore, more individual-based epidemiological studies should be conducted to confirm our results in the future. Thirdly, our study did not analyze the temporal trends of breast cancer burden attributable to risk factors, so further analyses are needed to investigate the variations of attributable burden across the BRICS-Plus countries over the past three decades.

In conclusion, breast cancer remains a serious health challenge among women across BRICS-Plus, and nearly half of the breast cancer incidence and death cases in the world contributed from overall BRICS-Plus, of which China, India and Pakistan were the highest. The age-standardized incidence and mortality rates increased in most of the BRICS-Plus countries over the past three decades, and the highest increase occurred in the SACU region. By APC analysis, breast cancer incidence, mortality and case fatality rates increased with advancing age in most of the BRICS-Plus countries, indicating older women might be the vulnerable population for breast cancer in these regions. The increasing cohort effect on incidence mortality risk revealed that the later birth cohorts were high-risk groups for the development of breast cancer. The rising burden of breast cancer in countries with lower socioeconomic status highlighted that more targeted and cost-effective measures should be strengthened for vulnerable populations in underdeveloped countries to screen and treat breast cancer.

## Data availability statement

Publicly available datasets were analyzed in this study. This data can be found here: The dataset analyzed during the current study are available in the Institute for Health Metrics and Evaluation (IHME): http://ghdx.healthdata.org/gbd-results-tool.

## Author contributions

CY supervised the study. SM and CY conceptualized the analysis. SM did the data analysis. SM and LL wrote the first draft of the paper. MI, N and JB reviewed and provided comments on the first draft. All authors reviewed the final draft, contributed to the article and approved the submitted version.
