# Supplementary material for: More recent insights into the breast cancer burden across BRICS-Plus: Health consequences in key nations with emerging economies using the global burden of disease study 2019
Source: Front Oncol. 2023 Jan 24;13:1100300. doi: 10.3389/fonc.2023.1100300 (PMC9902930; doi:10.3389/fonc.2023.1100300)
Supplement: Supplementary file 1 [file DataSheet_1.pdf]

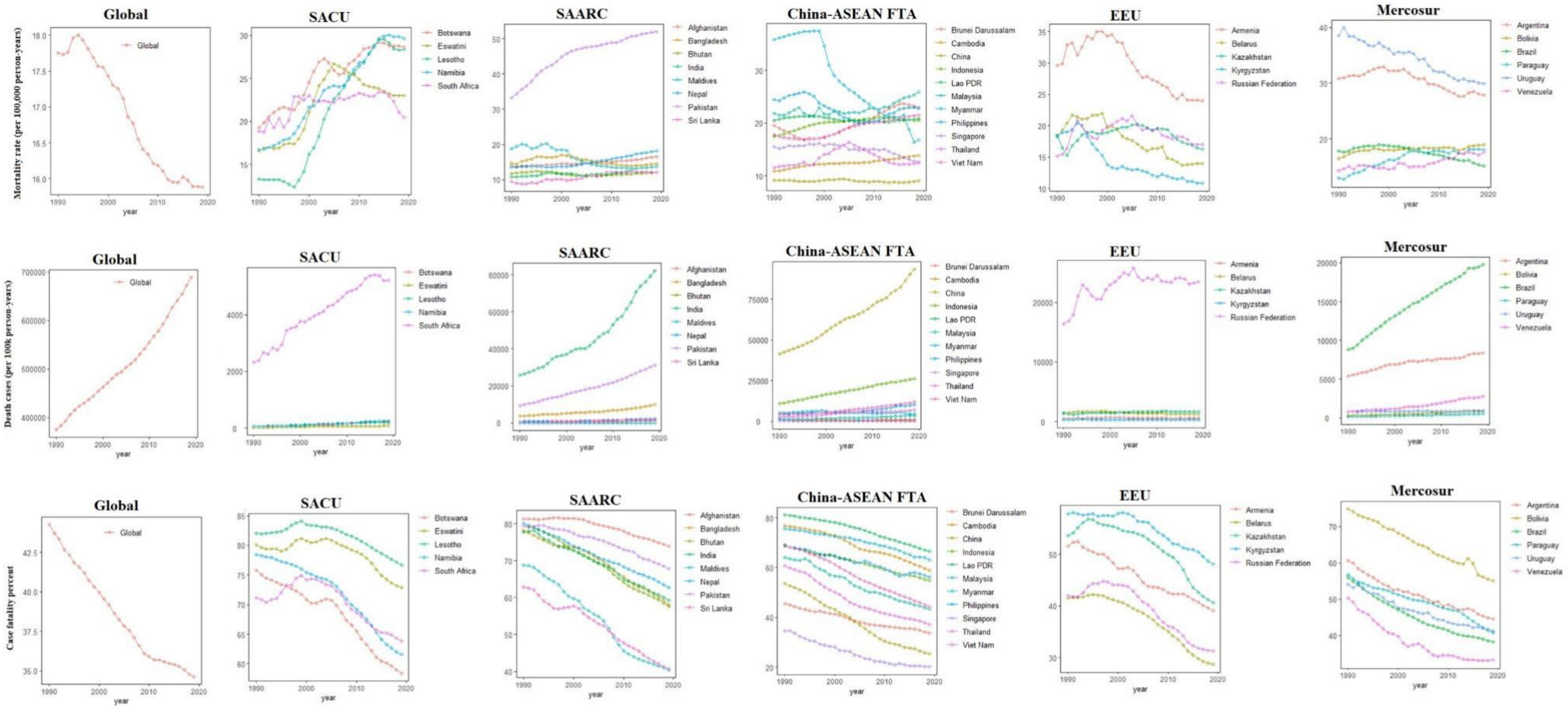

Figure S1. Age-standardised mortality rates, total number of death cases, and case fatality percent across BRICS-Plus and the world. Case fatality percent indicate the mortality to incidence ratio multiply by 100

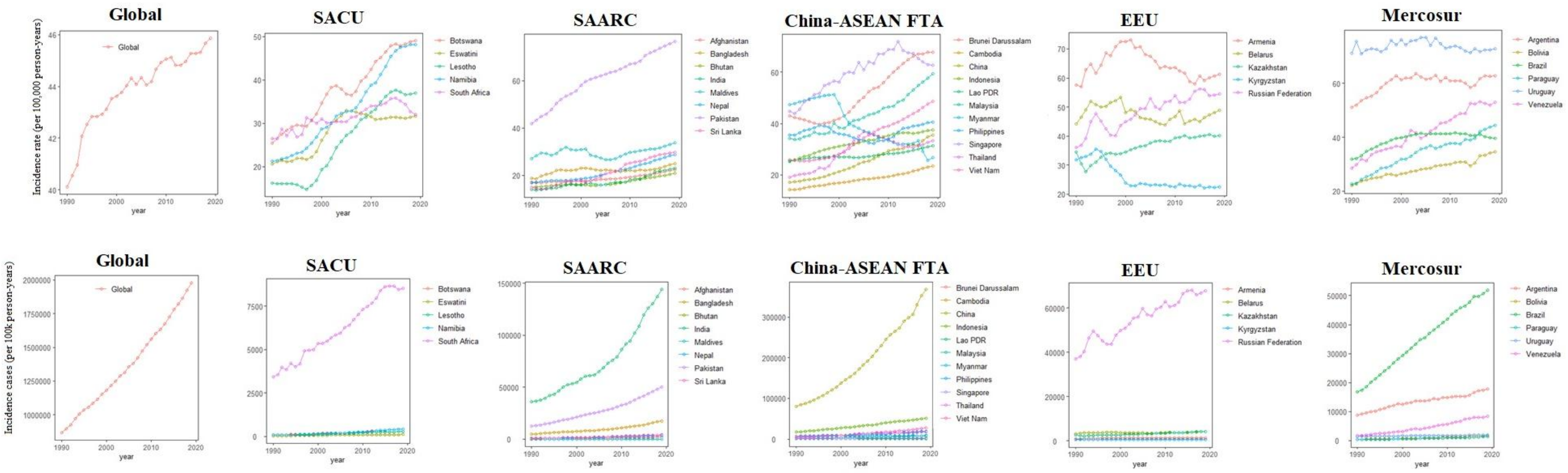

Figure S2. Age-standardised incidence rates and total number of incident cases across BRICS-Plus and the world

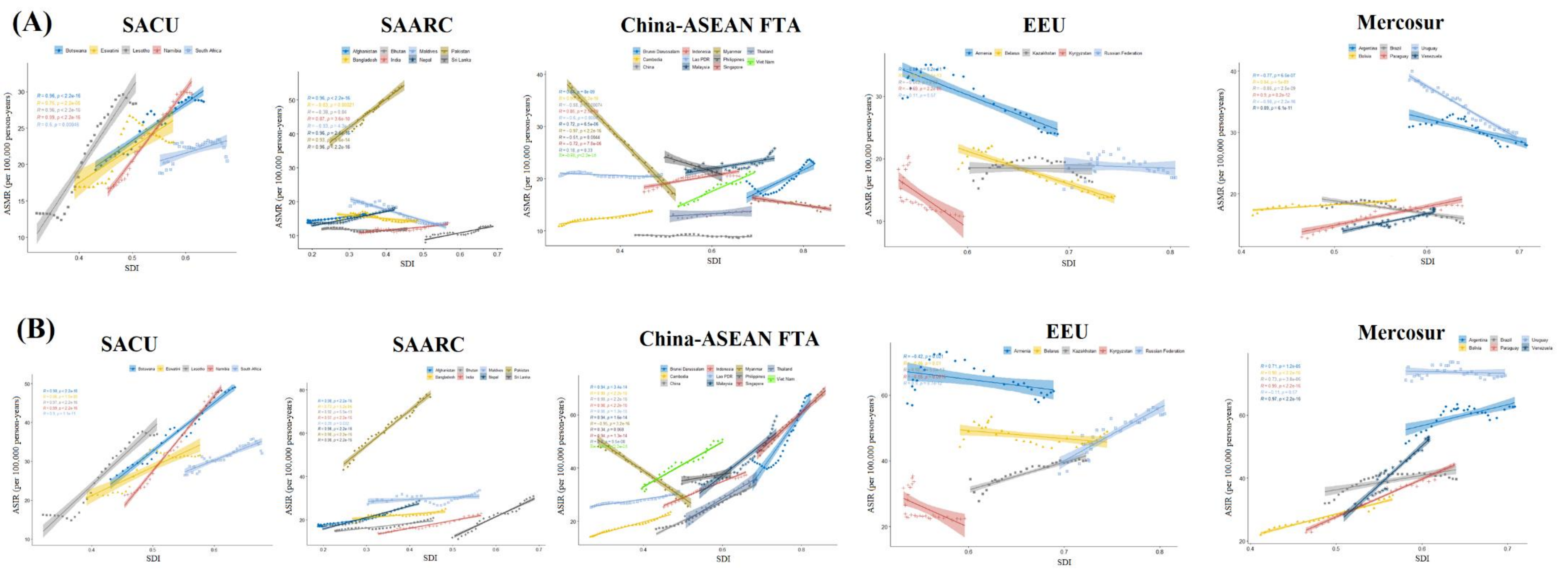

Figure S3. Time wise correlation between (A) Age-standardised mortality rate (ASMR) (B) Age-standardised incidence rate (ASIR) and country's sociodemographic index (SDI) from 1990 to 2019 across BRICS-Plus; R represent the correlation coefficient between SDI and breast cancer outcome across Brics-Plus region; SDI ranges from 0 (less developed) to 1 (most developed)

SACU

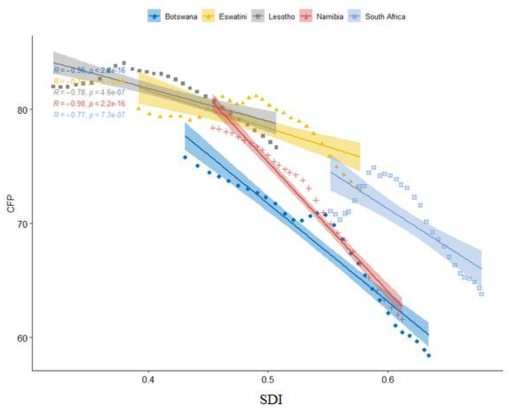

SAARC

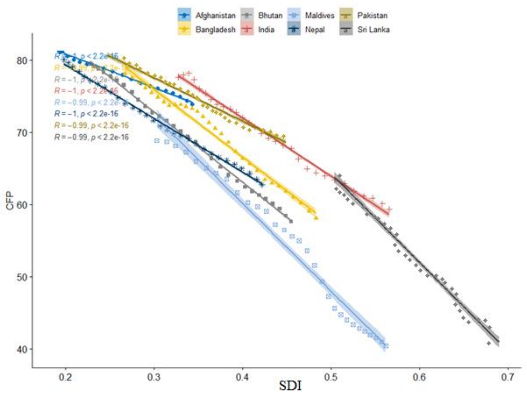

China-ASEAN FTA

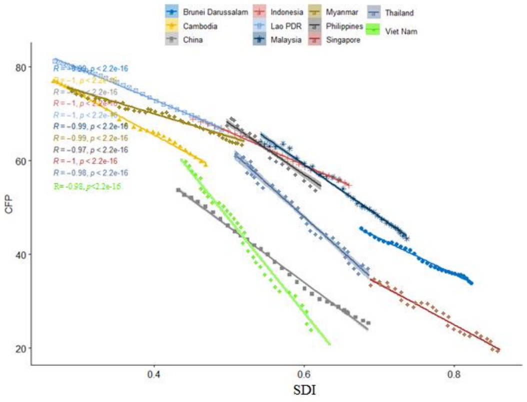

EEU

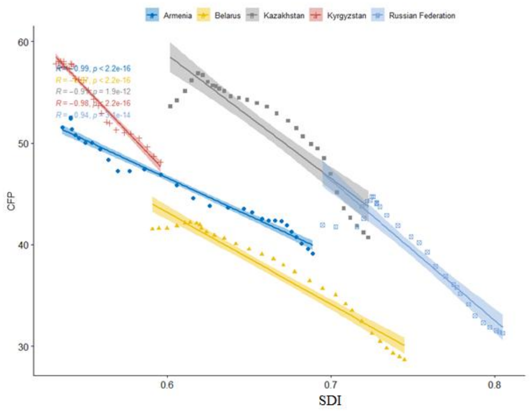

Mercosur

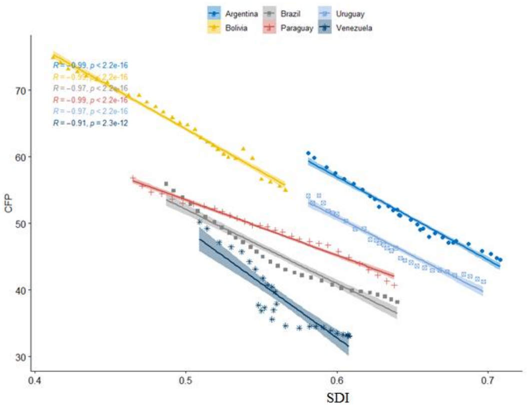

Figure S4. Time wise correlation between case fatality percent (CFP) and country's sociodemographic index (SDI) from 1990 to 2019 across BRICS-Plus; CFP indicate breast cancer age-standardised mortality to incidence ratio multiply by 100**;** R represent the correlation coefficient between SDI and CFP across BRICS-Plus region; SDI ranges from 0 (less developed) to 1 (most developed)
